# Probing Genome Maintenance Functions of human RECQ1

**DOI:** 10.5936/csbj.201303014

**Published:** 2013-10-18

**Authors:** Furqan Sami, Sudha Sharma

**Affiliations:** a Department of Biochemistry and Molecular Biology, College of Medicine, Howard University, 520 W Street, NW, Washington, DC 20059, USA

**Keywords:** Helicase, RecQ, DNA repair, DNA damage, genomic instability

## Abstract

The RecQ helicases are a highly conserved family of DNA-unwinding enzymes that play key roles in protecting the genome stability in all kingdoms of life. Human RecQ homologs include RECQ1, BLM, WRN, RECQ4, and RECQ5β. Although the individual RecQ-related diseases are characterized by a variety of clinical features encompassing growth defects (Bloom Syndrome and Rothmund Thomson Syndrome) to premature aging (Werner Syndrome), all these patients have a high risk of cancer predisposition. Here, we present an overview of recent progress towards elucidating functions of RECQ1 helicase, the most abundant but poorly characterized RecQ homolog in humans. Consistent with a conserved role in genome stability maintenance, deficiency of RECQ1 results in elevated frequency of spontaneous sister chromatid exchanges, chromosomal instability, increased DNA damage and greater sensitivity to certain genotoxic stress. Delineating what aspects of RECQ1 catalytic functions contribute to the observed cellular phenotypes, and how this is regulated is critical to establish its biological functions in DNA metabolism. Recent studies have identified functional specialization of RECQ1 in DNA repair; however, identification of fundamental similarities will be just as critical in developing a unifying theme for RecQ actions, allowing the functions revealed from studying one homolog to be extrapolated and generalized to other RecQ homologs.

## Introduction

Helicases are motor proteins that utilize the energy derived from nucleotide hydrolysis to disrupt double- or multi-stranded nucleic acids during the process of DNA replication, repair, and recombination, as well as RNA transcription, maturation, and translation. Helicases are recognized by the presence of conserved signature sequence motifs; all helicases possess sequences homologous to the Walker A and B boxes that are characteristic of NTP binding and/or hydrolyzing enzymes [1]. They do not display sequence specificity for unwinding, but instead exhibit preference for the type and structure of the nucleic acid substrate. DNA helicases facilitate unwinding either passively by binding and trapping single strand DNA, or they actively destabilize paired DNA in addition to binding the released single stranded DNA [1]. Unwinding is directional, either 3’-5’ or 5’-3’ with respect to the strand on which the helicase translocates.

## RecQ Helicase Family

**Figure 1:**
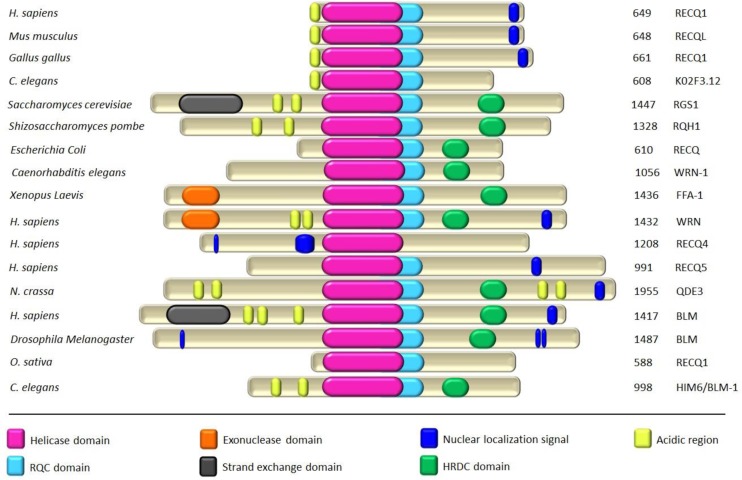
**Schematic representation of selected members of the RecQ-Like DNA helicases across species.** Members of the RecQ family have many structural motifs that are conserved from bacteria through humans. Besides the core helicase domain, most members possess RecQ C-terminal (RQC) and Helicase and RNase D C-terminal (HRDC) domains that mediate interactions with nucleic acids and proteins. A few RecQ proteins have acidic regions that are responsible for protein-protein interactions. WRN and FFA-1 proteins are unique in that they also contain an exonuclease domain. Total number of amino acids in each protein is indicated on the right and the full color scheme is indicated. Sequence of each protein in FASTA format was used as input to an online server called MyHits (http://myhits.isb-sib.ch/) for the mapping of various motifs in each protein.

The RecQ family represents one of the most highly conserved groups of 3’-5’ DNA helicases, and is named after the prototype *Escherichia coli* RecQ [2–5]. A single RecQ homolog exists in bacteria and yeast whereas higher eukaryotes possess multiple markedly conserved representatives (Figure 1).

The RecQ helicase family has 5 known homologs in the human genome: *RECQ1, WRN, BLM, RecQ4, and RecQ5β*. Mutations in three human RecQ helicase homologs WRN, BLM and RecQ4 are related to rare genetic disorders of Werner Syndrome, Bloom Syndrome, and Rothmund-Thomson/ RAPADILINO/Baller-Gerold Syndrome, respectively, all characterized by chromosomal instability and predisposition to cancer [6, 7]. The fact that these disorders are rare attests to the critical importance of these helicases in cellular DNA metabolism. The fact that the deficiency of individual RecQ helicases is manifested as clinically distinct syndromes supports the notion that human RecQ homologs function in distinct cellular processes. A systematic analysis of the molecular interactions and cellular functions of each RecQ homolog, and the comparison of similarities and differences among them, is likely to reveal aspects of RecQ functions that are important for genome maintenance.

## RECQ1 Helicase

**Figure 2:**
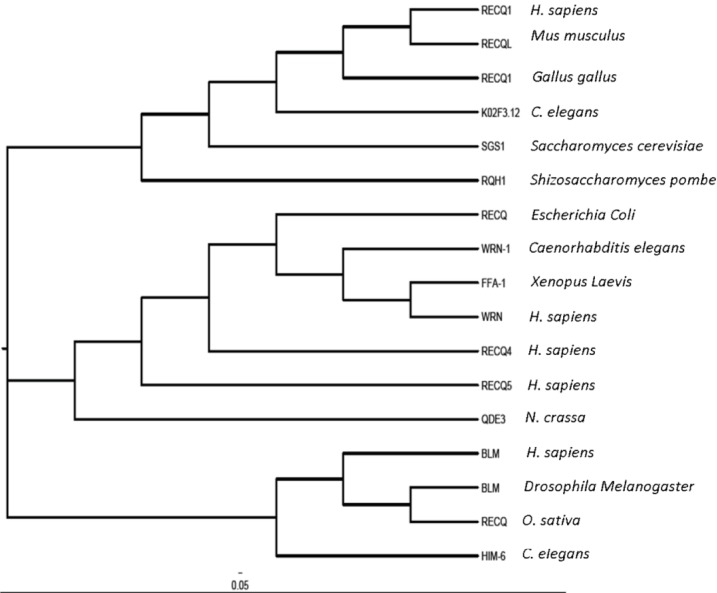
**Phylogenetic tree for the RecQ-Like proteins of DExH-Box helicase family.** Phylogenetic analysis of the selected RecQ DNA helicases was performed using clustalX 2.0 and the image was generated using FigTree version 1.4.0. The branches of the tree are abbreviated with codes and detailed with genus and species name on the right.

The *RECQ1* (also known as *RECQL* or *RECQL1*) gene resides on chromosome 12p12 and encodes a 649 amino acid protein with a molecular mass of 73 kDa [8–10]. RECQ1 protein is smallest of the human RecQ homologs, and shares maximum homology to the prototype *E. coli* RecQ. RECQ1 is a DNA-stimulated ATPase and helicase [10, 11]. Phylogenetic analysis of RECQ1 with closely related proteins reveals structural divergence (Figure 2).

A further insight into the anticipated biological roles of RECQ1 emanates from reviewing its structure-function relationship and molecular interactions.

### Biochemical activities and substrate specificities

RECQ1 unwinds DNA with a 3’-5’ polarity [12] and needs a 3′-single strand DNA tail to unwind the substrate [11]. RECQ1 unwinds standard duplex DNA substrates such as forked duplex, 3’-overhang or 3’-flap, 5’-flap, and synthetic replication fork structures; these substrates signify model replication and repair intermediates lacking single strand character in the 3’, 5’, or both arms adjacent to the DNA duplex [11, 13–16]. Apart from conventional helicase activity, RECQ1, like BLM and WRN, also promotes branch migration of Holliday junction (HJ) and D-loops in an ATP-dependent fashion [14–16]. RECQ1 unwinds three-stranded D-loop with either a distended single stranded 3’- or 5’-tail by releasing the invading third strand from D-loop structures although a D-loop with protruding single stranded 3’-tail is a preferred substrate for unwinding [14]. In contrast to BLM helicase, RECQ1 is unable to unwind a DNA-RNA hybrid, catalyze fork regression, or displace plasmid D-loops lacking a 3’-tail but can unwind four-armed synthetic HJ structures that lacked a homologous core [14–16]. Unlike other known branch migration proteins such as BLM helicase and RAD54 both of which show no significant preference in directionality of branch migration, RECQ1 specifically catalyzes unidirectional branch migration, which may be instrumental in specific disruption of toxic, nonproductive intermediates of homologous recombination (HR) during DNA double strand break (DSB) repair *in vivo* [17]. However, RECQ1 is unable to use its motor ATPase to strip RAD51 from DNA during HR repair [18]. RECQ1 is also incapable of displacing streptavidin from a biotinylated oligonucleotide [19]. Consistent with a 3’-5’ directionality of RECQ1 translocation on DNA, RECQ1 helicase activity is inhibited in a strand-specific manner by an alkyl phosphotriester modification to the sugar-phosphate backbone in the predicted translocating strand [20]. Moreover, the inability of RECQ1 to unwind G-quadruplex substrates differentiates this protein from other RecQ helicases including WRN, BLM, Sgs1, or *E. coli* RecQ [15, 21]. Specific functions of RECQ1 in DNA metabolism are not yet clearly understood but the reported disparity in helicase substrate preference suggests functional specialization.

In addition to DNA unwinding, RECQ1 promotes annealing of complementary single strand DNA in an ATP-independent manner [14]. ATP binding induces a conformational change in RECQ1 switching it from a strand-annealing protein to a DNA unwinding activity [14]. Further studies have suggested that distinct biochemical activities of RECQ1 are dictated by different oligomeric states modulated by single strand DNA and ATP binding [22]. Overall, strand-annealing appears to be an intrinsic property of the human RecQ family since it is conserved in WRN, BLM, RecQ4 and RecQ5β [23–25].

### Structural basis of biochemical functions

**Figure 3:**
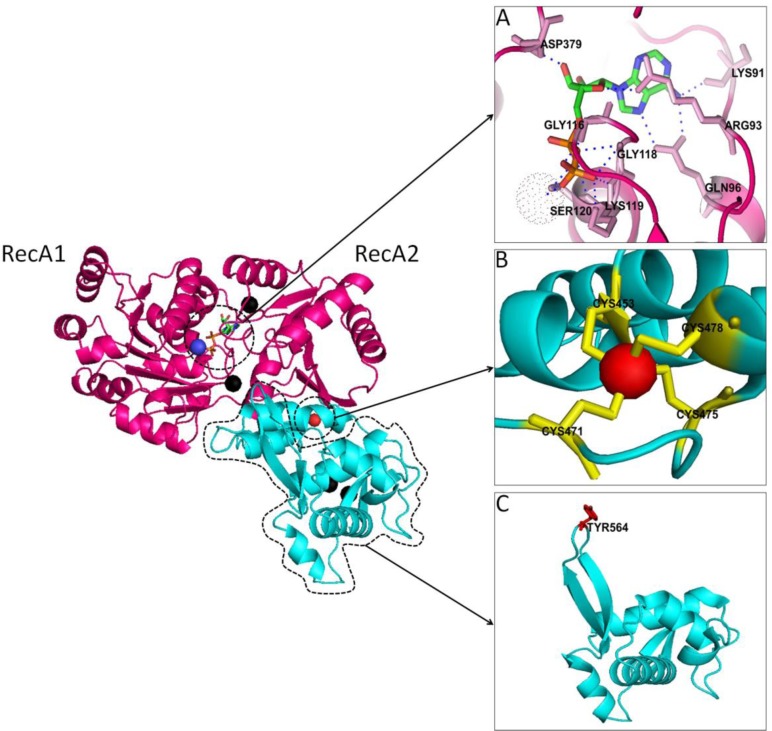
**Structural insight to the conserved helicase and RQC domains of human RECQ1 helicase (PDB ID: 2v1x).** Cartoon representation of a single RECQ1 molecule bound to ADP; color coding of conserved structural domains is consistent with the color scheme in Figure 1. Two RecA-like domains are denoted as RecA1 and RecA2. *A*. The nucleotide-binding pocket. ADP forms extensive contacts with RecA1 and less with RecA2; residues in contact with ADP are shown as sticks in pink color. *B*. RecQ-specific Zinc-binding module in RECQ1. A single Zn^2+^ ion is coordinated by four Cys residues positioned on two antiparallel α-helices. *C*. The winged-helix (WH) domain of RECQ1. β-hairpin is shown in the upper-left corner and the important aromatic residue Tyr 564 is highlighted in red color. The figure was generated using PyMOL (http://www.pymol.org/).

RecQ helicases share a centrally located helicase domain that couples nucleotide hydrolysis to DNA unwinding and defines the RecQ family (Figure 1). Other conserved domains include RQC (RecQ C-terminal) and HRDC ((helicase and RNaseD C-terminal), missing in RECQ1) domains, which are implicated in protein interactions and DNA binding [26–28]. The N- and C-terminal extensions in eukaryotic RecQ helicases are poorly conserved but are shown to mediate protein-protein interactions [29, 30]. Crystal structure of human RECQ1 protein lacking the first 48 and the last 33 amino acid residues (RECQ1^49-616^) was recently reported by Gileadi's group (Figure 3) [31]. Previously it was shown that the higher oligomeric structures formed by means of N-terminus region are responsible for DNA annealing activity of RECQ1 [15, 22]; consequently, truncated RECQ1^49-616^ failed to promote strand-annealing despite exhibiting unwinding of a forked-duplex comparable to the full-length RECQ1 [31]. Moreover, the N-terminal region of RECQ1, either direct or through the formation of higher order oligomers, also appears to be critical for the dissolution of HJs [15, 31]. Importantly, WRN protein was also shown to bind HJ and replication fork structures as an oligomer although whether this requires the N-terminal of WRN is not reported [32].

### RECQ1 Signature Helicase Domain

Core helicase domain of RECQ1 (amino acid residues 63-418) contains the seven signature motifs of superfamily 2 (SF2) helicases and harbors the ATP-binding pocket surrounded by highly conserved residues [3, 33]. Mutational analyses in RecQ homologs and other SF2 helicases have revealed the necessity of these amino acids for nucleotide binding as well as for functions related to nucleotide binding activity [34–36]. We and others have shown that ATP binding regulates the helicase and strand-annealing activities of RECQ1 [14, 22]. The overall fold of X-ray structure of RECQ1 bound to ADP and Mg^2+^ in the absence of nucleic acid was found to be comparable to other SF2 helicases having signature structure of two RecA-like domains containing the seven motifs, and retaining the general helicase fold (Figure 3) [31]. The relative orientation of the two RecA-like domains in multiple crystal forms of RECQ1 was found to be changed significantly suggesting a higher degree of flexibility between these two domains [31]. Previous studies have reported that *E. coli* RecQ shows slight relative rotation of the helicase domain in nucleotide-bound form compared to the unbound state and as a result of this rotation, motif I acquires an open conformation that allows the nucleotide to enter into its designated cavity [36]. Motif 0 in human RECQ1, located at the N-terminus of motif I is a RecQ-specific variant of the conserved Q motif in DEAD-box helicases and is implicated in ATP binding and hydrolysis [37]. The crystal structure of nucleotide-bound RECQ1 showed that the adenine moiety of ADP is hydrogen bonded to Gln96 within the motif 0 (Figure 3) [31]. Notably, amino acid substitution of Gln in Motif 0 of RecQ5β and BLM has been reported to reduce the ATPase and ATPase/helicase function, respectively [30, 34].

### RQC Domain of RECQ1

RQC domain in RECQ1 is composed of a Zn-binding module and a helix-turn-helix fold called winged-helix (WH) domain. The Zn-binding module of RQC domain is practically identical between bacterial and human enzymes; in RQC domain of human RECQ1, a Zn^2+^ ion is coordinated by four Cys residues positioned on two antiparallel α-helices (Figure 3) [31]. The Zn-binding domain of *E. coli* RecQ [38] and human BLM [39] are important in protein/DNA binding and protein folding. Missense mutations affecting the Cys residues of the Zn-binding pocket of BLM are found in Bloom syndrome patients [39]. The WH domain, also present in transcription factors such as CAP and hRFX1, and the human DNA repair protein AGT, acts as a DNA-binding motif [31, 33, 40]. Superimposition of the WH domains from *E. coli* RecQ, RECQ1 and WRN show an extraordinarily conserved fold [31]. Yet, the WH of RECQ1 interacts with significantly different regions of its core helicase and Zn-binding domains as compared to those of *E. coli* RecQ and the human WRN protein [31]. Furthermore, a unique tyrosine residue (Tyr564) at the tip of a β-hairpin structure within the WH domain of RECQ1 is suggested to control helicase activity to unwind a simple fork duplex independent of its DNA-dependent ATPase activity (Figure 3) [31]. Vindigni's group has reported that the β-hairpin in the WH domain of RECQ1 is essential for DNA unwinding and oligomer formation [41].

### Protein interactions of RECQ1

Table 1 enlists the known protein interactions of RECQ1 that are either unique or overlapping with those exhibited by other RecQ helicases. First identified protein interactions of RECQ1 were with the importin α homologs Rch1 and Qip1 [42]; Qip1 binds to the nuclear localization signal of RECQ1 (Figure 1) [43] and thus likely mediates its import to the nucleus where RECQ1 is primarily localized [44].

**Table 1:** Unique and redundant protein partners of RECQ1.

Interacting Proteins	RECQ1	WRN	BLM	RecQ4	RecQ5β
*RPA*	Yes [13]	Yes [45]	Yes [46]	Yes [47]	Yes [30]
*RAD51*	Yes [44]	Yes [48]	Yes [49]	Yes [50]	Yes [51]
*Top3α*	Yes [52]		Yes [52]		Yes [53]
*EXOI*	Yes [54]	Yes [55]	Yes [56]		
*PARP1*	Yes [57]	Yes [58]		Yes [59]	
*MSH2/6*	Yes [54]	Yes [60]	Yes [61]		
*MLH1-PMS2*	Yes [54]	Yes [60]	Yes [62]		
*Ku70/80*	Yes [63]	Yes [64]			
*Importin alpha1/KPNA2/Rch1*	Yes [42]				
*Importin alpha3/KPNA4/Qip1*	Yes [43]				
*Histone H2A type 1-A*	Yes [65]				
*Histone H2A type 2-A*	Yes [65]				
*Histone H2B type 1-L*	Yes [65]				
*Histone 3.2*	Yes [65]				

One of the conserved physical and functional interactions of human RecQ helicases is with the single strand DNA binding protein, RPA [3]. Stimulation of WRN or BLM catalyzed DNA unwinding by RPA requires physical interaction with the helicase [66]. RPA interacts with RECQ1 and stimulates its DNA unwinding activity [13] while inhibiting strand-annealing [14]. Physical interaction of RPA heterotrimer with RECQ1 is mediated through the RPA70 subunit [13] which was also shown to be sufficient for physical and functional interaction with WRN [66]. Interaction site of RPA on RECQ1 has not yet been mapped, but RECQ1 does contain an acidic region similar to that in WRN which is reported to mediate interaction of WRN with RPA (Figure 1) [66]. RECQ1 also associates with Topoisomerase 3α [67] and RAD51 [44]; but the functional implication of these interactions remains to be elucidated [24]. Both BLM [68] and RecQ5β [51] dissociate RAD51 from DNA *in vitro* serving as anti-recombinase activities; whereas BLM-Topoisomerase 3α complex is exclusively important in dissolution of double HJs [69]. RECQ1 interacts physically and functionally with mismatch repair proteins (MLH1 /PMS2, MSH2/MSH6, and EXO-1) which are also involved in regulation of genetic recombination [54]. The mismatch recognition complex MSH2/MSH6 stimulates RECQ1 helicase activity whereas RECQ1 stimulates the incision activity of human EXO-1 [54]. Thus an anti-recombination function of RECQ1 may involve suppression of homeologous recombination in conjunction with mismatch repair factors that specifically bind base pair mismatches [24, 25]. In recent years, EXO-1 has emerged to be critical in helping cells deal with stalled replication forks [70–72] and interaction of RECQ1 with EXO-1 may also be important in this capacity. Another key protein partner of RECQ1 appears to be PARP-1 [57, 65]. We first identified a direct protein interaction of RECQ1 with PARP-1 and demonstrated that RECQ1-PARP-1-RPA associate in a common protein complex [57]. A conceivable biological function of this complex might be to regulate opposing activities of RECQ1 that are known to be regulated by RPA and ATP [14]. Given the ability of PARP-1 to modulate cellular ATP pools [73], interaction with PARP-1 may be important in providing an appropriate microenvironment to regulate dual activities of RECQ1 as necessary for the given cellular context. Moreover, RECQ1 and PARP-1 are also capable of interacting with the common components of mismatch repair system [54, 74] indicating a possible role in the suppression of homeologous recombination between diverged sequences. Molecular basis for the elevated sister chromatid exchanges (SCEs) in RECQ1 deficiency is not yet understood, but its interactions with mismatch repair proteins (MLH1/PMS2, MSH2/MSH6, and EXO-1) and PARP-1 may be relevant in this regard. Vindigni's lab has recently identified an exclusive role of RECQ1-PARP-1 interaction in the process of replication restart following Topoisomerase 1 (TOP1) inhibition [65]. They have demonstrated that RECQ1 preferentially catalyzes fork reversal and promotes resetting of replication forks *in vitro*. Following TOP1 inhibition by Camptothecin (CPT) treatment, the poly(ADP)ribosylation activity of PARP-1 inhibits fork reversal by RECQ1 *in vivo* [65]. Thus, RECQ1-PARP-1 complex stabilizes regressed forks until repair of the TOP1 cleavage complex is complete thus preventing premature restart of regressed forks [65]. PARP activity is not required in RECQ1-depleted cells as they cannot promote fork restoration in the absence of RECQ1 [65]; these cells likely employ HR for restart of replication following CPT treatment. Other known members of RECQ1 complex with RPA and PARP-1 are Ku70/80 [63, 65] along with certain nucleosomal histones [65]. Interestingly, RECQ1 was also found to be present with PARP-1 and Ku80 in a multi-protein complex of APLF (APTX-PNK-Like Factor) which is implicated in recruitment of non-homologous end joining (NHEJ) proteins at DSBs [75–78]. Subsequently, we have shown that RECQ1 exhibits a direct physical interaction with the Ku70/80 subunit of DNA-PK complex and modulates *in vitro* end joining of DSBs [63]. Collectively, these studies have provided a framework for understanding the biological roles of RECQ1 though it is still premature to propose a comprehensive model for RECQ1 in the context of its catalytic functions and protein interactions. Identification of additional constituents of the RECQ1-containing protein complexes and elucidation of how they modulate its biochemical activities will be critical to establish precise roles of RECQ1 in pathways of replication restart and DNA strand break repair.

### RECQ1 in genome maintenance

Reported cellular phenotypes of RECQ1-deficiency in mouse and humans implicate unique requirement of RECQ1 in genome stability maintenance [24, 44, 79]. Despite the lack of a phenotypic defect in unstressed RECQ1 knockout mice, primary embryonic fibroblasts from RECQ1 knockout mice display chromosomal instability [79]; and increased chromosomal instability is also observed upon depletion of RECQ1 in human cells [44]. Cellular deficiency of RECQ1 is characterized by spontaneously elevated SCEs [24] which are reminiscent of recombinogenic structures proposed to arise during replication restart following fork collapse [80]. Indeed, RECQ1-deficient cells accumulate DNA damage and display increased sensitivity to DNA damaging agents that induce stalled and collapsed replication forks [44, 65, 81, 82]. Furthermore, RECQ1-deficiency in mice [79] or human cells [44] results in heightened sensitivity to ionizing radiation, which also causes oxidative DNA damage.

Our recent findings provide preliminary evidence for a unique role of RECQ1 in repair of oxidative DNA damage [57]. When cells are exposed to hydrogen peroxide (H_2_O_2_), RECQ1 is among the first RecQ proteins to arrive on chromatin and remains associated for the period of time required for the repair of strand breaks [57]. Remarkably, the chromatin localization of RECQ1 is more robust and rapid than that of WRN helicase which has been shown to function in oxidative DNA damage repair [57]. It is plausible that the activities of these RecQ proteins are assigned to dedicated pathways or sub-pathways of oxidative DNA damage repair [83, 84]. RECQ1 and its protein partners may be part of the DNA damage response by localizing to sites of oxidative lesions where they execute catalytic functions, alone or in concert. Consistent with this notion, purified recombinant RECQ1 catalyzes unwinding of duplex DNA containing oxidative base lesion such as thymine glycol, and the presence of RPA stimulates DNA unwinding by RECQ1 when the thymine glycol is positioned in the nontranslocating strand for the helicase [85]. RECQ1-depleted cells rely on PARP activity for the repair of H_2_O_2_-induced DNA damage and when RECQ1 is deficient, these lesions are possibly repaired by an alternative mechanism that involves increased activation of PARP [57]. In contrast, WRN-deficient cells fail to activate PARP in response to oxidative damage [57]. These observations suggest a novel and non-overlapping role of RECQ1 in exogenously induced-oxidative DNA damage repair via modulation of PARP-1; but a direct role of RECQ1 in specific pathway of oxidative DNA damage repair remains to be elucidated.

PARP-1 is known to bind to DNA strand breaks and subsequently synthesizes and transfers poly(ADP-ribose) polymers to itself and various nuclear proteins [86]. BLM or WRN-depleted cells exhibit constitutively hyperactivated PARP, hypersensitivity to PARP inhibitors, and are defective in HR [87]. In contrast, depletion of RECQ1 by itself does not lead to PARP hyperactivation or enhanced sensitivity to PARP inhibitor [57]. Moreover, RECQ1-depleted cells are not compromised in their ability to repair I-SceI-induced DSB by homology directed repair [57]. In addition to HR, DSBs are repaired by NHEJ mediated by Ku70/80, the DNA-PKcs protein kinase, and the complex consisting of DNA ligase IV, XRCC4 and XLF [88]. Our more recent data indicates that RECQ1 and Ku70/80 co-bind a linear DNA and the DNA binding by Ku70/80 is modulated by the presence of RECQ1 [63]. Recent models of DSB repair propose that Ku binds DNA ends first and is subsequently released through the DNA end processing activities contributed by MRN complex, CtIP, and EXO-1 [89, 90]. Ku70/80 inhibits EXO-1-mediated DSB resection *in vivo* [91] and DNA end resection of the forked duplex substrate *in vitro* [92]. The ability of RECQ1 to bind and unwind a Ku-bound forked DNA duplex relatively efficiently and its known interaction with EXO-1 suggests that RECQ1 may enable EXO-1 to overcome Ku inhibition and thereby modulate the pathway choice for DSB repair [92].

It is important to note that HR mechanisms involved in repairing classical two ended DSBs are distinct from those provoked by replication stress [93]. Notably, RECQ1, along with RecQ4, is an integral component of replication complex in unperturbed dividing cells [94]. Association of RECQ1 with replication origins during normal replication is significantly enhanced when cells encounter replication stress [82, 94]. Consistent with this, recombinant RECQ1 binds and unwinds model replication forks [14], and promotes strand exchange on stalled replication forks *in vitro* [81]. RECQ1-depletion results in increased sensitivity to aphidicolin, diminished checkpoint activation in response to replication stress, and chromosomal instability [82]. Chromatin immunoprecipitation experiments revealed that RECQ1 is preferentially enriched at two major fragile sites, FRA3B and FRA16D, where replication forks have stalled *in vivo* following aphidicolin treatment [82]. Common fragile sites are randomly distributed slow replicating genomic regions that are particularly vulnerable to replication stress and expressed as site-specific gaps or breaks on metaphase chromosomes after partial inhibition of DNA synthesis [95]. Fragile sites often coincide with chromosomal breakpoints in tumors [95, 96] and stalled replication forks at common fragile sites are believed to be a major cause of genomic instability [95, 97]. Consistent with its demonstrated catalytic functions [65, 81], recruitment of RECQ1 at fragile sites indicates that RECQ1 facilitates repair of stalled or collapsed replication forks and preserves genome integrity [82]. These results also implicate RECQ1 in mechanisms underlying common fragile site instability in cancer. Remarkably, RECQ1 is overexpressed in transformed cells [98] and in many clinical cancer samples compared to matched normal samples indicating potential target for cancer therapy [82, 99, 100]. Single nucleotide polymorphisms of RECQ1 have been associated with reduced survival in pancreatic cancer [101, 102]. RECQ1 expression is critical for the growth and proliferation of a variety of cancer cells [44, 103]; this has also been demonstrated using xenograft models [104]. It is conceivable that cancer cells are overtly dependent upon RECQ1 activities to cope with replication-induced DNA damage during rapid cell division; in normal cells, RECQ1 can act as a tumor suppressor by facilitating DNA repair and preventing mutations.

## Summary and Outlook

Recent advances in structural analyses and identification of novel molecular interactions has provided a valuable foundation to explore unique and overlapping functions of RECQ1. In particular, importance of RECQ1 in repair of DNA damage in the context of replication stress has become increasingly more apparent. DNA lesions induced by replication stress occur predominantly in early replicating and actively transcribed gene clusters [105]. Given the presence of RECQ1 at replication origins, it will be insightful to investigate whether RECQ1 has a role in preventing transcription-associated genetic instability. Molecular functions of RecQ and other DNA helicases in cancer are becoming more prominent [106]. Reported overexpression of RECQ1 in a variety of clinical cancer merits systematic investigation of clinicopathological correlation. While a disease association remains to be discovered, unique requirement of RECQ1 in suppressing genomic instability proposes that a defect in RECQ1 may be linked to cancer predisposition disorders that are distinct from known RecQ-diseases. A greater understanding of the molecular and cellular functions of RECQ1 is essential to establish its role in genome maintenance and explore its translational potential.
